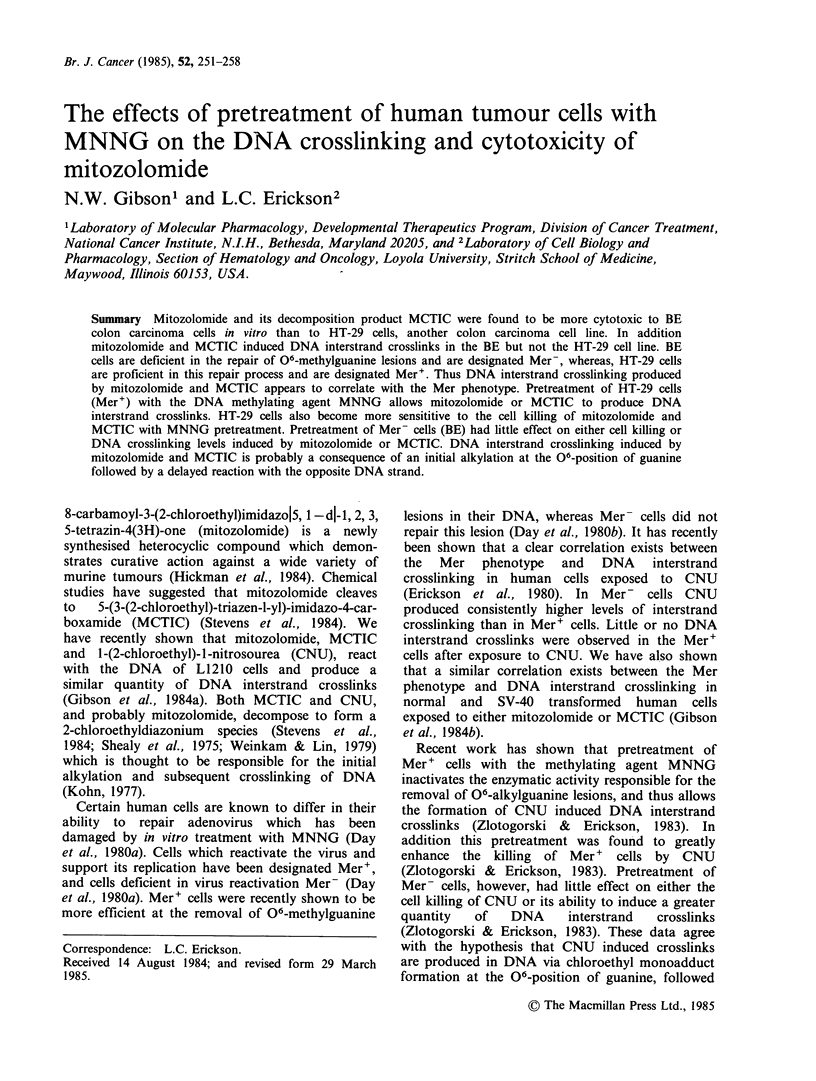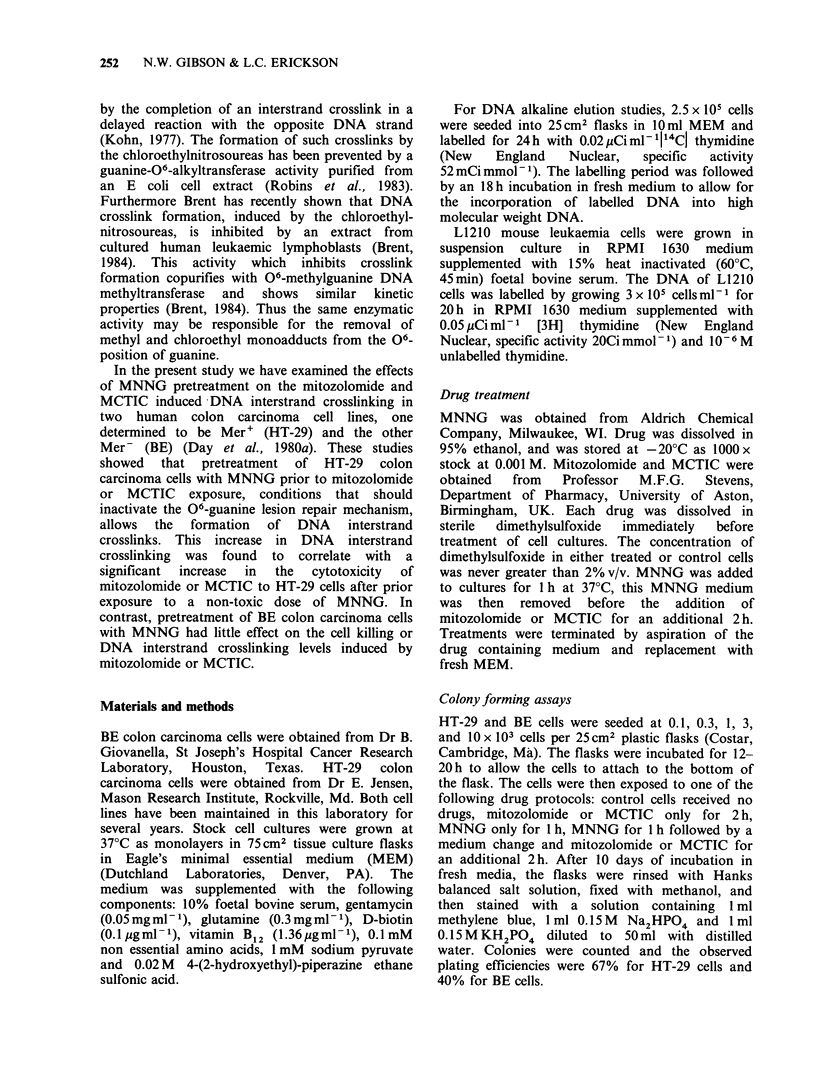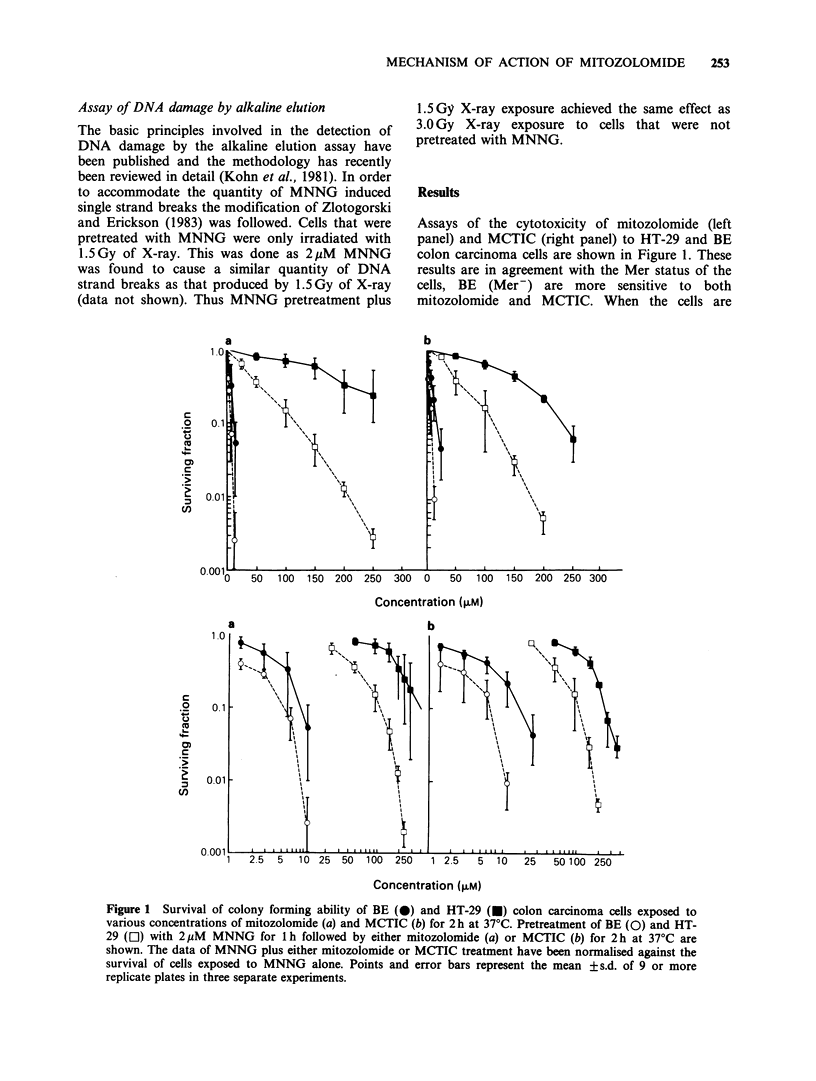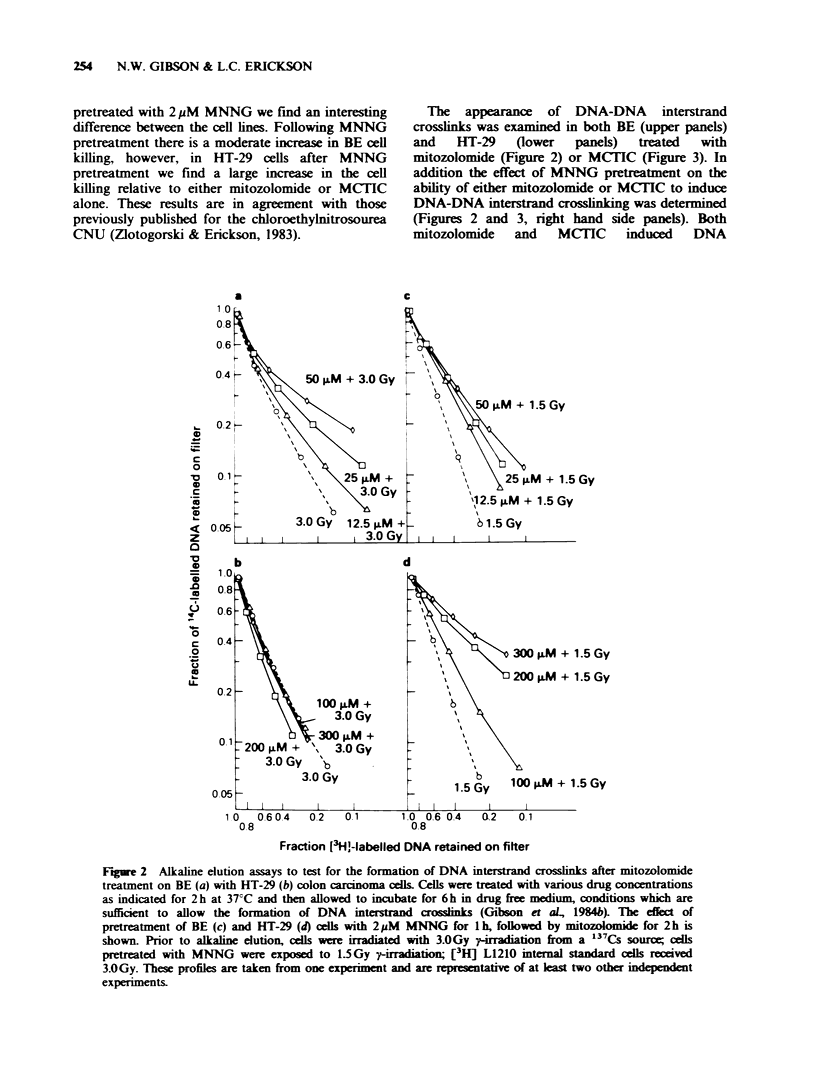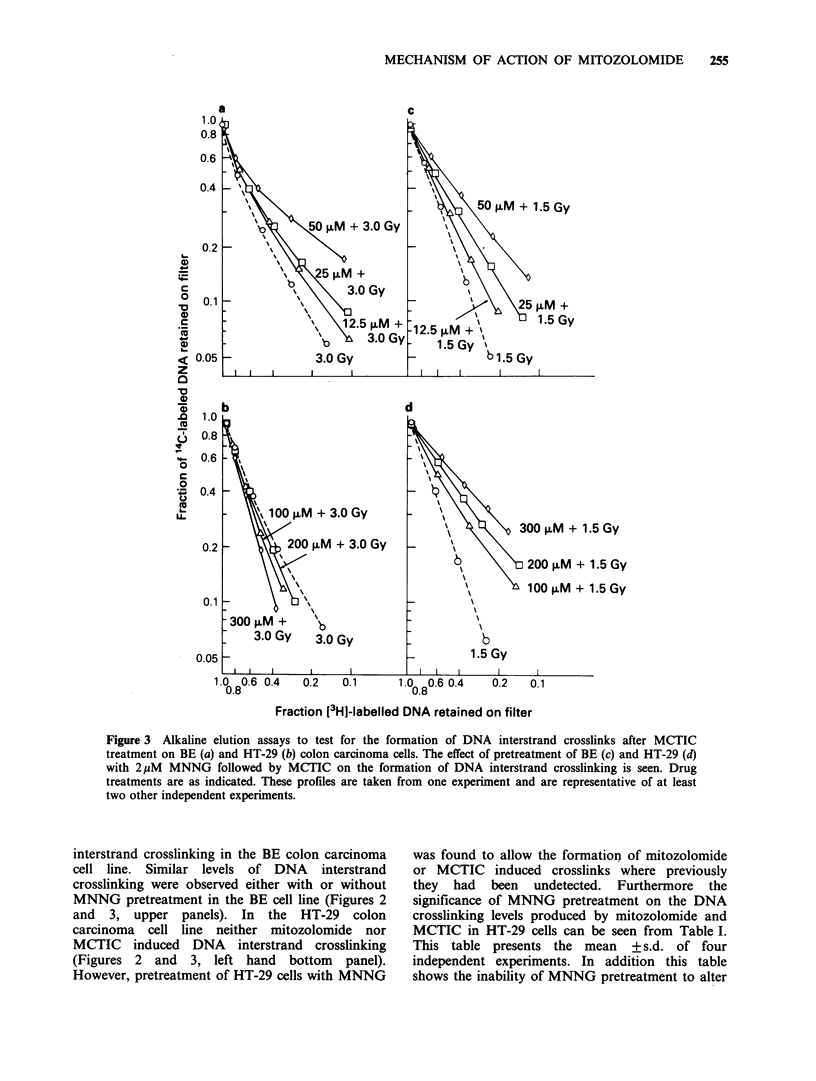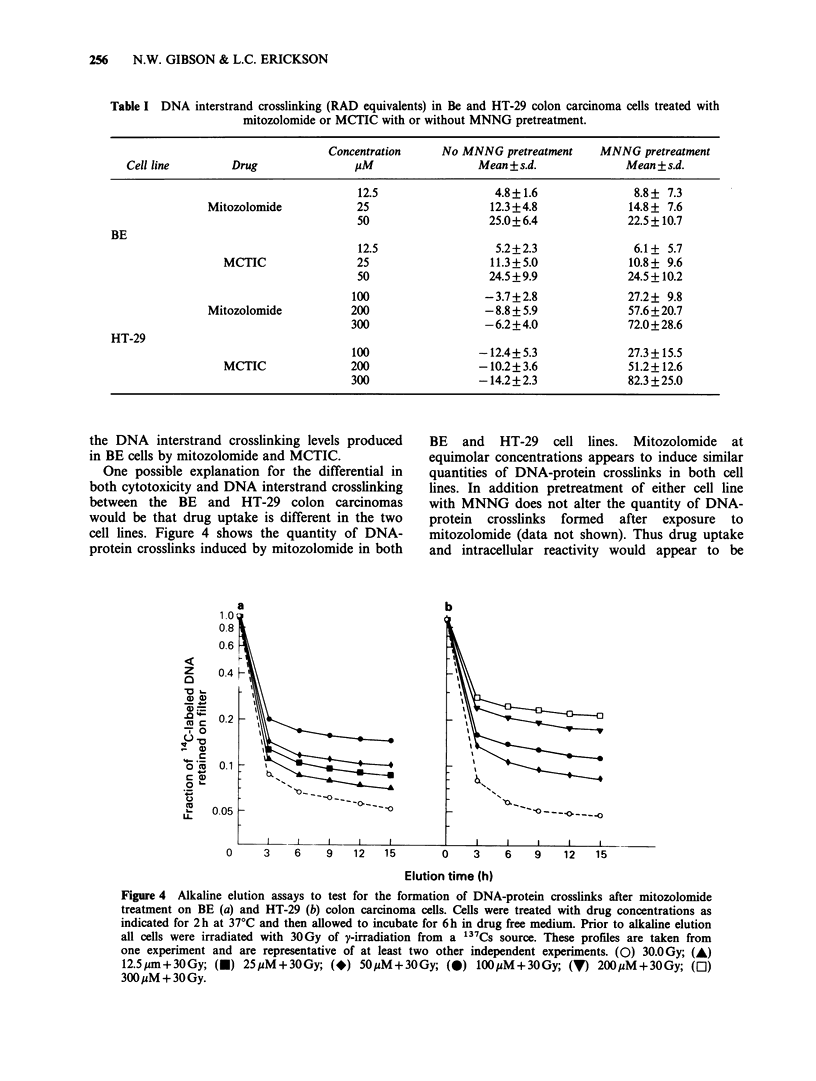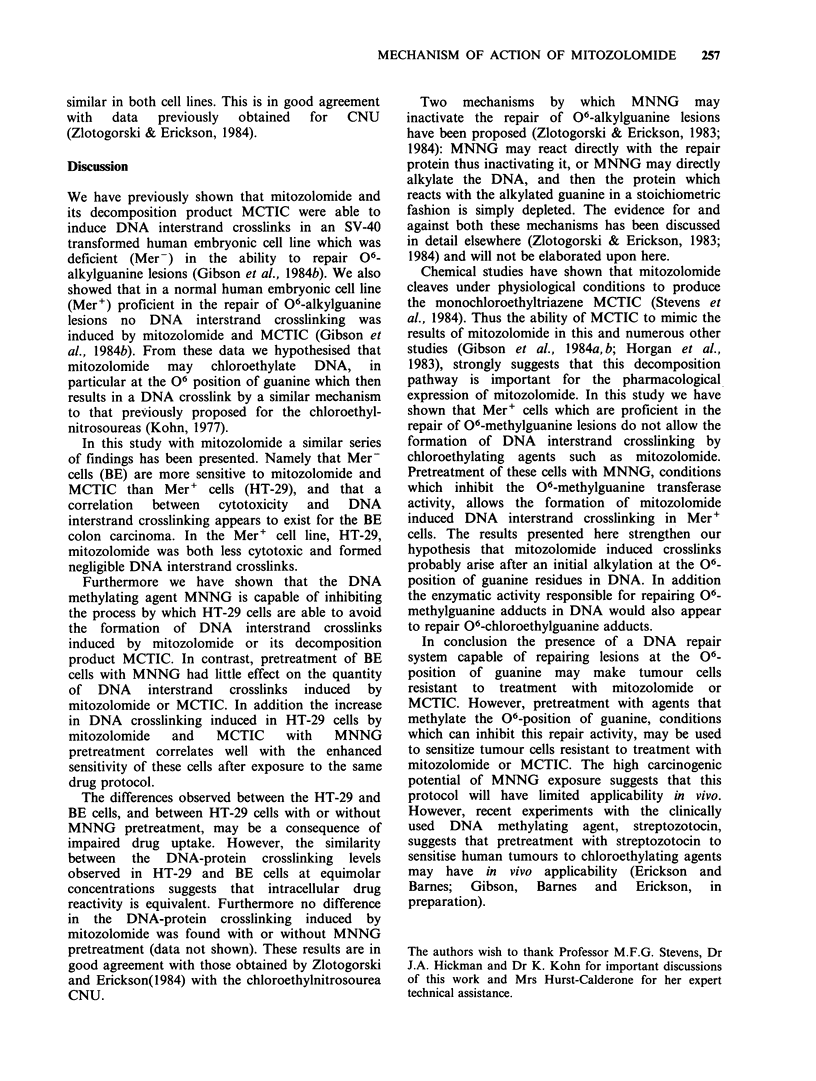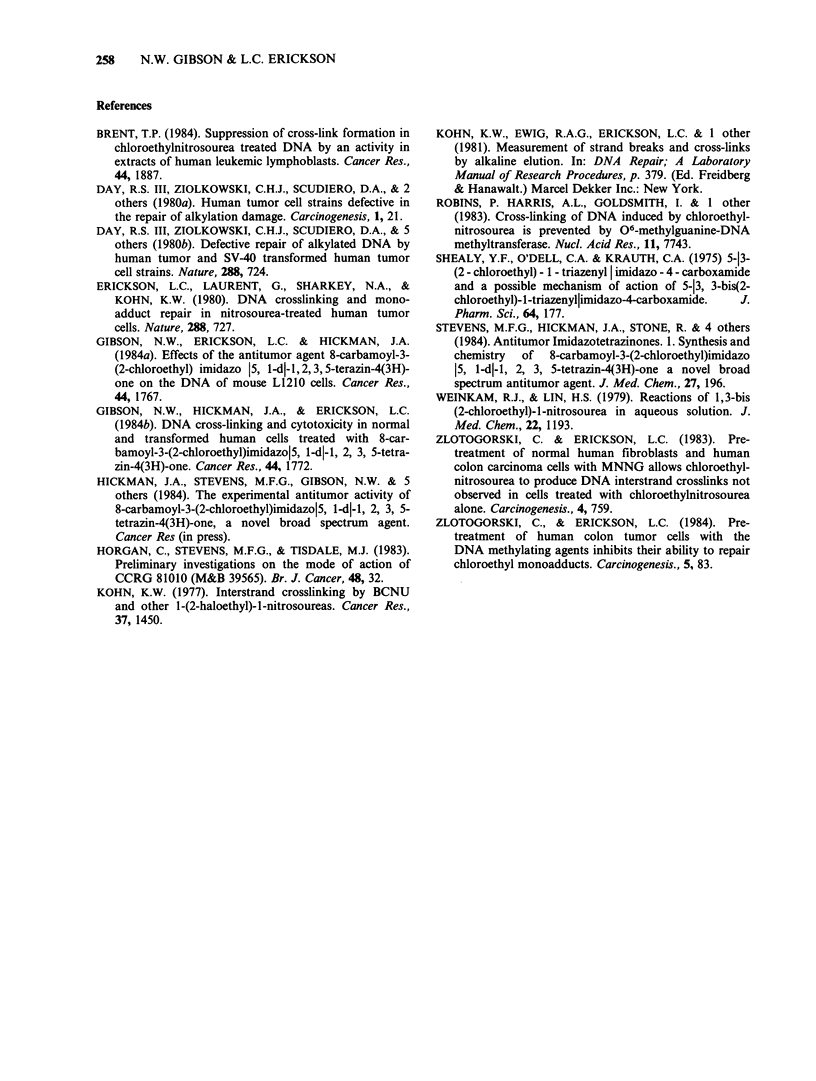# The effects of pretreatment of human tumour cells with MNNG on the DNA crosslinking and cytotoxicity of mitozolomide.

**DOI:** 10.1038/bjc.1985.185

**Published:** 1985-08

**Authors:** N. W. Gibson, L. C. Erickson

## Abstract

Mitozolomide and its decomposition product MCTIC were found to be more cytotoxic to BE colon carcinoma cells in vitro than to HT-29 cells, another colon carcinoma cell line. In addition mitozolomide and MCTIC induced DNA interstrand crosslinks in the BE but not the HT-29 cell line. BE cells are deficient in the repair of O6-methylguanine lesions and are designated Mer-, whereas, HT-29 cells are proficient in this repair process and are designated Mer+. Thus DNA interstrand crosslinking produced by mitozolomide and MCTIC appears to correlate with the Mer phenotype. Pretreatment of HT-29 cells (Mer+) with the DNA methylating agent MNNG allows mitozolomide or MCTIC to produce DNA interstrand crosslinks. HT-29 cells also become more sensitive to the cell killing of mitozolomide and MCTIC with MNNG pretreatment. Pretreatment of Mer- cells (BE) had little effect on either cell killing or DNA crosslinking levels induced by mitozolomide or MCTIC. DNA interstrand crosslinking induced by mitozolomide and MCTIC is probably a consequence of an initial alkylation at the O6-position of guanine followed by a delayed reaction with the opposite DNA strand.


*Br. J. Cancer* (1985), **52**, 251–258

### The effects of pretreatment of human tumour cells with MNNG on the DNA crosslinking and cytotoxicity of mitozolomide

N.W. Gibson[1] and L.C. Erickson[2]

[1]*Laboratory of Molecular Pharmacology, Developmental Therapeutics Program, Division of Cancer Treatment, National Cancer Institute, N.I.H., Bethesda, Maryland 20205, and* [2]*Laboratory of Cell Biology and Pharmacology, Section of Hematology and Oncology, Loyola University, Stritch School of Medicine, Maywood, Illinois 60153, USA.*

**Summary** Mitozolomide and its decomposition product MCTIC were found to be more cytotoxic to BE colon carcinoma cells *in vitro* than to HT-29 cells, another colon carcinoma cell line. In addition mitozolomide and MCTIC induced DNA interstrand crosslinks in the BE but not the HT-29 cell line. BE cells are deficient in the repair of O$^6$-methylguanine lesions and are designated Mer$^-$, whereas, HT-29 cells are proficient in this repair process and are designated Mer$^+$. Thus DNA interstrand crosslinking produced by mitozolomide and MCTIC appears to correlate with the Mer phenotype. Pretreatment of HT-29 cells (Mer$^+$) with the DNA methylating agent MNNG allows mitozolomide or MCTIC to produce DNA interstrand crosslinks. HT-29 cells also become more sensitive to the cell killing of mitozolomide and MCTIC with MNNG pretreatment. Pretreatment of Mer$^-$ cells (BE) had little effect on either cell killing or DNA crosslinking levels induced by mitozolomide or MCTIC. DNA interstrand crosslinking induced by mitozolomide and MCTIC is probably a consequence of an initial alkylation at the O$^6$-position of guanine followed by a delayed reaction with the opposite DNA strand.

8-carbamoyl-3-(2-chloroethyl)imidazo|5, 1 – d|-1, 2, 3, 5-tetrazin-4(3H)-one (mitozolomide) is a newly synthesised heterocyclic compound which demonstrates curative action against a wide variety of murine tumours (Hickman *et al.*, 1984). Chemical studies have suggested that mitozolomide cleaves to 5-(3-(2-chloroethyl)-triazen-l-yl)-imidazo-4-carboxamide (MCTIC) (Stevens *et al.*, 1984). We have recently shown that mitozolomide, MCTIC and 1-(2-chloroethyl)-1-nitrosourea (CNU), react with the DNA of L1210 cells and produce a similar quantity of DNA interstrand crosslinks (Gibson *et al.*, 1984a). Both MCTIC and CNU, and probably mitozolomide, decompose to form a 2-chloroethyldiazonium species (Stevens *et al.*, 1984; Shealy *et al.*, 1975; Weinkam & Lin, 1979) which is thought to be responsible for the initial alkylation and subsequent crosslinking of DNA (Kohn, 1977).

Certain human cells are known to differ in their ability to repair adenovirus which has been damaged by *in vitro* treatment with MNNG (Day *et al.*, 1980*a*). Cells which reactivate the virus and support its replication have been designated Mer$^+$, and cells deficient in virus reactivation Mer$^-$ (Day *et al.*, 1980*a*). Mer$^+$ cells were recently shown to be more efficient at the removal of O$^6$-methylguanine lesions in their DNA, whereas Mer$^-$ cells did not repair this lesion (Day *et al.*, 1980*b*). It has recently been shown that a clear correlation exists between the Mer phenotype and DNA interstrand crosslinking in human cells exposed to CNU (Erickson *et al.*, 1980). In Mer$^-$ cells CNU produced consistently higher levels of interstrand crosslinking than in Mer$^+$ cells. Little or no DNA interstrand crosslinks were observed in the Mer$^+$ cells after exposure to CNU. We have also shown that a similar correlation exists between the Mer phenotype and DNA interstrand crosslinking in normal and SV-40 transformed human cells exposed to either mitozolomide or MCTIC (Gibson *et al.*, 1984*b*).

Recent work has shown that pretreatment of Mer$^+$ cells with the methylating agent MNNG inactivates the enzymatic activity responsible for the removal of O$^6$-alkylguanine lesions, and thus allows the formation of CNU induced DNA interstrand crosslinks (Zlotogorski & Erickson, 1983). In addition this pretreatment was found to greatly enhance the killing of Mer$^+$ cells by CNU (Zlotogorski & Erickson, 1983). Pretreatment of Mer$^-$ cells, however, had little effect on either the cell killing of CNU or its ability to induce a greater quantity of DNA interstrand crosslinks (Zlotogorski & Erickson, 1983). These data agree with the hypothesis that CNU induced crosslinks are produced in DNA via chloroethyl monoadduct formation at the O$^6$-position of guanine, followed

Correspondence: L.C. Erickson.
Received 14 August 1984; and revised form 29 March 1985.

© The Macmillan Press Ltd., 1985

by the completion of an interstrand crosslink in a delayed reaction with the opposite DNA strand (Kohn, 1977). The formation of such crosslinks by the chloroethylnitrosoureas has been prevented by a guanine-$O^6$-alkyltransferase activity purified from an E coli cell extract (Robins *et al.*, 1983). Furthermore Brent has recently shown that DNA crosslink formation, induced by the chloroethylnitrosoureas, is inhibited by an extract from cultured human leukaemic lymphoblasts (Brent, 1984). This activity which inhibits crosslink formation copurifies with $O^6$-methylguanine DNA methyltransferase and shows similar kinetic properties (Brent, 1984). Thus the same enzymatic activity may be responsible for the removal of methyl and chloroethyl monoadducts from the $O^6$-position of guanine.

In the present study we have examined the effects of MNNG pretreatment on the mitozolomide and MCTIC induced DNA interstrand crosslinking in two human colon carcinoma cell lines, one determined to be $Mer^+$ (HT-29) and the other $Mer^-$ (BE) (Day *et al.*, 1980*a*). These studies showed that pretreatment of HT-29 colon carcinoma cells with MNNG prior to mitozolomide or MCTIC exposure, conditions that should inactivate the $O^6$-guanine lesion repair mechanism, allows the formation of DNA interstrand crosslinks. This increase in DNA interstrand crosslinking was found to correlate with a significant increase in the cytotoxicity of mitozolomide or MCTIC to HT-29 cells after prior exposure to a non-toxic dose of MNNG. In contrast, pretreatment of BE colon carcinoma cells with MNNG had little effect on the cell killing or DNA interstrand crosslinking levels induced by mitozolomide or MCTIC.

### Materials and methods

BE colon carcinoma cells were obtained from Dr B. Giovanella, St Joseph's Hospital Cancer Research Laboratory, Houston, Texas. HT-29 colon carcinoma cells were obtained from Dr E. Jensen, Mason Research Institute, Rockville, Md. Both cell lines have been maintained in this laboratory for several years. Stock cell cultures were grown at 37°C as monolayers in $75\,cm^2$ tissue culture flasks in Eagle's minimal essential medium (MEM) (Dutchland Laboratories, Denver, PA). The medium was supplemented with the following components: 10% foetal bovine serum, gentamycin ($0.05\,mg\,ml^{-1}$), glutamine ($0.3\,mg\,ml^{-1}$), D-biotin ($0.1\,\mu g\,ml^{-1}$), vitamin $B_{12}$ ($1.36\,\mu g\,ml^{-1}$), 0.1 mM non essential amino acids, 1 mM sodium pyruvate and 0.02 M 4-(2-hydroxyethyl)-piperazine ethane sulfonic acid.

For DNA alkaline elution studies, $2.5 \times 10^5$ cells were seeded into $25\,cm^2$ flasks in 10 ml MEM and labelled for 24 h with $0.02\,\mu Ci\,ml^{-1}$ $|^{14}C|$ thymidine (New England Nuclear, specific activity $52\,mCi\,mmol^{-1}$). The labelling period was followed by an 18 h incubation in fresh medium to allow for the incorporation of labelled DNA into high molecular weight DNA.

L1210 mouse leukaemia cells were grown in suspension culture in RPMI 1630 medium supplemented with 15% heat inactivated (60°C, 45 min) foetal bovine serum. The DNA of L1210 cells was labelled by growing $3 \times 10^5$ cells $ml^{-1}$ for 20 h in RPMI 1630 medium supplemented with $0.05\,\mu Ci\,ml^{-1}$ [3H] thymidine (New England Nuclear, specific activity $20 Ci\,mmol^{-1}$) and $10^{-6}$ M unlabelled thymidine.

### *Drug treatment*

MNNG was obtained from Aldrich Chemical Company, Milwaukee, WI. Drug was dissolved in 95% ethanol, and was stored at −20°C as 1000 × stock at 0.001 M. Mitozolomide and MCTIC were obtained from Professor M.F.G. Stevens, Department of Pharmacy, University of Aston, Birmingham, UK. Each drug was dissolved in sterile dimethylsulfoxide immediately before treatment of cell cultures. The concentration of dimethylsulfoxide in either treated or control cells was never greater than 2% v/v. MNNG was added to cultures for 1 h at 37°C, this MNNG medium was then removed before the addition of mitozolomide or MCTIC for an additional 2 h. Treatments were terminated by aspiration of the drug containing medium and replacement with fresh MEM.

### *Colony forming assays*

HT-29 and BE cells were seeded at 0.1, 0.3, 1, 3, and $10 \times 10^3$ cells per $25\,cm^2$ plastic flasks (Costar, Cambridge, Mà). The flasks were incubated for 12–20 h to allow the cells to attach to the bottom of the flask. The cells were then exposed to one of the following drug protocols: control cells received no drugs, mitozolomide or MCTIC only for 2 h, MNNG only for 1 h, MNNG for 1 h followed by a medium change and mitozolomide or MCTIC for an additional 2 h. After 10 days of incubation in fresh media, the flasks were rinsed with Hanks balanced salt solution, fixed with methanol, and then stained with a solution containing 1 ml methylene blue, 1 ml 0.15 M $Na_2HPO_4$ and 1 ml 0.15 M $KH_2PO_4$ diluted to 50 ml with distilled water. Colonies were counted and the observed plating efficiencies were 67% for HT-29 cells and 40% for BE cells.

*Assay of DNA damage by alkaline elution*

The basic principles involved in the detection of DNA damage by the alkaline elution assay have been published and the methodology has recently been reviewed in detail (Kohn *et al.*, 1981). In order to accommodate the quantity of MNNG induced single strand breaks the modification of Zlotogorski and Erickson (1983) was followed. Cells that were pretreated with MNNG were only irradiated with 1.5 Gy of X-ray. This was done as 2 $\mu$M MNNG was found to cause a similar quantity of DNA strand breaks as that produced by 1.5 Gy of X-ray (data not shown). Thus MNNG pretreatment plus 1.5 Gy X-ray exposure achieved the same effect as 3.0 Gy X-ray exposure to cells that were not pretreated with MNNG.

### Results

Assays of the cytotoxicity of mitozolomide (left panel) and MCTIC (right panel) to HT-29 and BE colon carcinoma cells are shown in Figure 1. These results are in agreement with the Mer status of the cells, BE (Mer$^-$) are more sensitive to both mitozolomide and MCTIC. When the cells are

**Figure 1** Survival of colony forming ability of BE (●) and HT-29 (■) colon carcinoma cells exposed to various concentrations of mitozolomide (*a*) and MCTIC (*b*) for 2 h at 37°C. Pretreatment of BE (○) and HT-29 (□) with 2 $\mu$M MNNG for 1 h followed by either mitozolomide (*a*) or MCTIC (*b*) for 2 h at 37°C are shown. The data of MNNG plus either mitozolomide or MCTIC treatment have been normalised against the survival of cells exposed to MNNG alone. Points and error bars represent the mean ±s.d. of 9 or more replicate plates in three separate experiments.

pretreated with 2 $\mu$M MNNG we find an interesting difference between the cell lines. Following MNNG pretreatment there is a moderate increase in BE cell killing, however, in HT-29 cells after MNNG pretreatment we find a large increase in the cell killing relative to either mitozolomide or MCTIC alone. These results are in agreement with those previously published for the chloroethylnitrosourea CNU (Zlotogorski & Erickson, 1983).

The appearance of DNA-DNA interstrand crosslinks was examined in both BE (upper panels) and HT-29 (lower panels) treated with mitozolomide (Figure 2) or MCTIC (Figure 3). In addition the effect of MNNG pretreatment on the ability of either mitozolomide or MCTIC to induce DNA-DNA interstrand crosslinking was determined (Figures 2 and 3, right hand side panels). Both mitozolomide and MCTIC induced DNA

**Figure 2** Alkaline elution assays to test for the formation of DNA interstrand crosslinks after mitozolomide treatment on BE (*a*) with HT-29 (*b*) colon carcinoma cells. Cells were treated with various drug concentrations as indicated for 2 h at 37°C and then allowed to incubate for 6 h in drug free medium, conditions which are sufficient to allow the formation of DNA interstrand crosslinks (Gibson *et al.*, 1984*b*). The effect of pretreatment of BE (*c*) and HT-29 (*d*) cells with 2 $\mu$M MNNG for 1 h, followed by mitozolomide for 2 h is shown. Prior to alkaline elution, cells were irradiated with 3.0 Gy $\gamma$-irradiation from a $^{137}$Cs source; cells pretreated with MNNG were exposed to 1.5 Gy $\gamma$-irradiation; [$^{3}$H] L1210 internal standard cells received 3.0 Gy. These profiles are taken from one experiment and are representative of at least two other independent experiments.

**Figure 3** Alkaline elution assays to test for the formation of DNA interstrand crosslinks after MCTIC treatment on BE (*a*) and HT-29 (*b*) colon carcinoma cells. The effect of pretreatment of BE (*c*) and HT-29 (*d*) with 2 μM MNNG followed by MCTIC on the formation of DNA interstrand crosslinking is seen. Drug treatments are as indicated. These profiles are taken from one experiment and are representative of at least two other independent experiments.

interstrand crosslinking in the BE colon carcinoma cell line. Similar levels of DNA interstrand crosslinking were observed either with or without MNNG pretreatment in the BE cell line (Figures 2 and 3, upper panels). In the HT-29 colon carcinoma cell line neither mitozolomide nor MCTIC induced DNA interstrand crosslinking (Figures 2 and 3, left hand bottom panel). However, pretreatment of HT-29 cells with MNNG was found to allow the formation of mitozolomide or MCTIC induced crosslinks where previously they had been undetected. Furthermore the significance of MNNG pretreatment on the DNA crosslinking levels produced by mitozolomide and MCTIC in HT-29 cells can be seen from Table I. This table presents the mean ±s.d. of four independent experiments. In addition this table shows the inability of MNNG pretreatment to alter

**Table I** DNA interstrand crosslinking (RAD equivalents) in Be and HT-29 colon carcinoma cells treated with mitozolomide or MCTIC with or without MNNG pretreatment.

| *Cell line* | *Drug* | *Concentration μM* | *No MNNG pretreatment Mean±s.d.* | *MNNG pretreatment Mean±s.d.* |
|---|---|---|---|---|
| BE | Mitozolomide | 12.5 | 4.8±1.6 | 8.8± 7.3 |
| | | 25 | 12.3±4.8 | 14.8± 7.6 |
| | | 50 | 25.0±6.4 | 22.5±10.7 |
| | MCTIC | 12.5 | 5.2±2.3 | 6.1± 5.7 |
| | | 25 | 11.3±5.0 | 10.8± 9.6 |
| | | 50 | 24.5±9.9 | 24.5±10.2 |
| HT-29 | Mitozolomide | 100 | −3.7±2.8 | 27.2± 9.8 |
| | | 200 | −8.8±5.9 | 57.6±20.7 |
| | | 300 | −6.2±4.0 | 72.0±28.6 |
| | MCTIC | 100 | −12.4±5.3 | 27.3±15.5 |
| | | 200 | −10.2±3.6 | 51.2±12.6 |
| | | 300 | −14.2±2.3 | 82.3±25.0 |

the DNA interstrand crosslinking levels produced in BE cells by mitozolomide and MCTIC.

One possible explanation for the differential in both cytotoxicity and DNA interstrand crosslinking between the BE and HT-29 colon carcinomas would be that drug uptake is different in the two cell lines. Figure 4 shows the quantity of DNA-protein crosslinks induced by mitozolomide in both BE and HT-29 cell lines. Mitozolomide at equimolar concentrations appears to induce similar quantities of DNA-protein crosslinks in both cell lines. In addition pretreatment of either cell line with MNNG does not alter the quantity of DNA-protein crosslinks formed after exposure to mitozolomide (data not shown). Thus drug uptake and intracellular reactivity would appear to be

**Figure 4** Alkaline elution assays to test for the formation of DNA-protein crosslinks after mitozolomide treatment on BE (*a*) and HT-29 (*b*) colon carcinoma cells. Cells were treated with drug concentrations as indicated for 2 h at 37°C and then allowed to incubate for 6 h in drug free medium. Prior to alkaline elution all cells were irradiated with 30 Gy of γ-irradiation from a $^{137}$Cs source. These profiles are taken from one experiment and are representative of at least two other independent experiments. (○) 30.0 Gy; (▲) 12.5 μm + 30 Gy; (■) 25 μM + 30 Gy; (◆) 50 μM + 30 Gy; (●) 100 μM + 30 Gy; (▼) 200 μM + 30 Gy; (□) 300 μM + 30 Gy.

similar in both cell lines. This is in good agreement with data previously obtained for CNU (Zlotogorski & Erickson, 1984).

### Discussion

We have previously shown that mitozolomide and its decomposition product MCTIC were able to induce DNA interstrand crosslinks in an SV-40 transformed human embryonic cell line which was deficient ($Mer^-$) in the ability to repair $O^6$-alkylguanine lesions (Gibson *et al.*, 1984*b*). We also showed that in a normal human embryonic cell line ($Mer^+$) proficient in the repair of $O^6$-alkylguanine lesions no DNA interstrand crosslinking was induced by mitozolomide and MCTIC (Gibson *et al.*, 1984*b*). From these data we hypothesised that mitozolomide may chloroethylate DNA, in particular at the $O^6$ position of guanine which then results in a DNA crosslink by a similar mechanism to that previously proposed for the chloroethylnitrosoureas (Kohn, 1977).

In this study with mitozolomide a similar series of findings has been presented. Namely that $Mer^-$ cells (BE) are more sensitive to mitozolomide and MCTIC than $Mer^+$ cells (HT-29), and that a correlation between cytotoxicity and DNA interstrand crosslinking appears to exist for the BE colon carcinoma. In the $Mer^+$ cell line, HT-29, mitozolomide was both less cytotoxic and formed negligible DNA interstrand crosslinks.

Furthermore we have shown that the DNA methylating agent MNNG is capable of inhibiting the process by which HT-29 cells are able to avoid the formation of DNA interstrand crosslinks induced by mitozolomide or its decomposition product MCTIC. In contrast, pretreatment of BE cells with MNNG had little effect on the quantity of DNA interstrand crosslinks induced by mitozolomide or MCTIC. In addition the increase in DNA crosslinking induced in HT-29 cells by mitozolomide and MCTIC with MNNG pretreatment correlates well with the enhanced sensitivity of these cells after exposure to the same drug protocol.

The differences observed between the HT-29 and BE cells, and between HT-29 cells with or without MNNG pretreatment, may be a consequence of impaired drug uptake. However, the similarity between the DNA-protein crosslinking levels observed in HT-29 and BE cells at equimolar concentrations suggests that intracellular drug reactivity is equivalent. Furthermore no difference in the DNA-protein crosslinking induced by mitozolomide was found with or without MNNG pretreatment (data not shown). These results are in good agreement with those obtained by Zlotogorski and Erickson(1984) with the chloroethylnitrosourea CNU.

Two mechanisms by which MNNG may inactivate the repair of $O^6$-alkylguanine lesions have been proposed (Zlotogorski & Erickson, 1983; 1984): MNNG may react directly with the repair protein thus inactivating it, or MNNG may directly alkylate the DNA, and then the protein which reacts with the alkylated guanine in a stoichiometric fashion is simply depleted. The evidence for and against both these mechanisms has been discussed in detail elsewhere (Zlotogorski & Erickson, 1983; 1984) and will not be elaborated upon here.

Chemical studies have shown that mitozolomide cleaves under physiological conditions to produce the monochloroethyltriazene MCTIC (Stevens *et al.*, 1984). Thus the ability of MCTIC to mimic the results of mitozolomide in this and numerous other studies (Gibson *et al.*, 1984*a,b*; Horgan *et al.*, 1983), strongly suggests that this decomposition pathway is important for the pharmacological expression of mitozolomide. In this study we have shown that $Mer^+$ cells which are proficient in the repair of $O^6$-methylguanine lesions do not allow the formation of DNA interstrand crosslinking by chloroethylating agents such as mitozolomide. Pretreatment of these cells with MNNG, conditions which inhibit the $O^6$-methylguanine transferase activity, allows the formation of mitozolomide induced DNA interstrand crosslinking in $Mer^+$ cells. The results presented here strengthen our hypothesis that mitozolomide induced crosslinks probably arise after an initial alkylation at the $O^6$-position of guanine residues in DNA. In addition the enzymatic activity responsible for repairing $O^6$-methylguanine adducts in DNA would also appear to repair $O^6$-chloroethylguanine adducts.

In conclusion the presence of a DNA repair system capable of repairing lesions at the $O^6$-position of guanine may make tumour cells resistant to treatment with mitozolomide or MCTIC. However, pretreatment with agents that methylate the $O^6$-position of guanine, conditions which can inhibit this repair activity, may be used to sensitize tumour cells resistant to treatment with mitozolomide or MCTIC. The high carcinogenic potential of MNNG exposure suggests that this protocol will have limited applicability *in vivo*. However, recent experiments with the clinically used DNA methylating agent, streptozotocin, suggests that pretreatment with streptozotocin to sensitise human tumours to chloroethylating agents may have *in vivo* applicability (Erickson and Barnes; Gibson, Barnes and Erickson, in preparation).

The authors wish to thank Professor M.F.G. Stevens, Dr J.A. Hickman and Dr K. Kohn for important discussions of this work and Mrs Hurst-Calderone for her expert technical assistance.

### References

BRENT, T.P. (1984). Suppression of cross-link formation in chloroethylnitrosourea treated DNA by an activity in extracts of human leukemic lymphoblasts. *Cancer Res.*, **44**, 1887.

DAY, R.S. III, ZIOLKOWSKI, C.H.J., SCUDIERO, D.A., & 2 others (1980*a*). Human tumor cell strains defective in the repair of alkylation damage. *Carcinogenesis*, **1**, 21.

DAY, R.S. III, ZIOLKOWSKI, C.H.J., SCUDIERO, D.A., & 5 others (1980*b*). Defective repair of alkylated DNA by human tumor and SV-40 transformed human tumor cell strains. *Nature*, **288**, 724.

ERICKSON, L.C., LAURENT, G., SHARKEY, N.A., & KOHN, K.W. (1980). DNA crosslinking and mono-adduct repair in nitrosourea-treated human tumor cells. *Nature*, **288**, 727.

GIBSON, N.W., ERICKSON, L.C. & HICKMAN, J.A. (1984*a*). Effects of the antitumor agent 8-carbamoyl-3-(2-chloroethyl) imidazo |5, 1-d|-1, 2, 3, 5-terazin-4(3H)-one on the DNA of mouse L1210 cells. *Cancer Res.*, **44**, 1767.

GIBSON, N.W., HICKMAN, J.A., & ERICKSON, L.C. (1984*b*). DNA cross-linking and cytotoxicity in normal and transformed human cells treated with 8-carbamoyl-3-(2-chloroethyl)imidazo|5, 1-d|-1, 2, 3, 5-tetrazin-4(3H)-one. *Cancer Res.*, **44**, 1772.

HICKMAN, J.A., STEVENS, M.F.G., GIBSON, N.W. & 5 others (1984). The experimental antitumor activity of 8-carbamoyl-3-(2-chloroethyl)imidazo|5, 1-d|-1, 2, 3, 5-tetrazin-4(3H)-one, a novel broad spectrum agent. *Cancer Res* (in press).

HORGAN, C., STEVENS, M.F.G., & TISDALE, M.J. (1983). Preliminary investigations on the mode of action of CCRG 81010 (M&B 39565). *Br. J. Cancer*, **48**, 32.

KOHN, K.W. (1977). Interstrand crosslinking by BCNU and other 1-(2-haloethyl)-1-nitrosoureas. *Cancer Res.*, **37**, 1450.

KOHN, K.W., EWIG, R.A.G., ERICKSON, L.C. & 1 other (1981). Measurement of strand breaks and cross-links by alkaline elution. In: *DNA Repair; A Laboratory Manual of Research Procedures*, *p.* 379. (Ed. Freidberg & Hanawalt.) Marcel Dekker Inc.: New York.

ROBINS, P. HARRIS, A.L., GOLDSMITH, I. & 1 other (1983). Cross-linking of DNA induced by chloroethylnitrosourea is prevented by $O^6$-methylguanine-DNA methyltransferase. *Nucl. Acid Res.*, **11**, 7743.

SHEALY, Y.F., O'DELL, C.A. & KRAUTH, C.A. (1975) 5-|3-(2-chloroethyl)-1-triazenyl|imidazo-4-carboxamide and a possible mechanism of action of 5-|3, 3-bis(2-chloroethyl)-1-triazenyl|imidazo-4-carboxamide. *J. Pharm. Sci.*, **64**, 177.

STEVENS, M.F.G., HICKMAN, J.A., STONE, R. & 4 others (1984). Antitumor Imidazotetrazinones. 1. Synthesis and chemistry of 8-carbamoyl-3-(2-chloroethyl)imidazo |5, 1-d|-1, 2, 3, 5-tetrazin-4(3H)-one a novel broad spectrum antitumor agent. *J. Med. Chem.*, **27**, 196.

WEINKAM, R.J., & LIN, H.S. (1979). Reactions of 1,3-bis (2-chloroethyl)-1-nitrosourea in aqueous solution. *J. Med. Chem.*, **22**, 1193.

ZLOTOGORSKI, C. & ERICKSON, L.C. (1983). Pretreatment of normal human fibroblasts and human colon carcinoma cells with MNNG allows chloroethylnitrosourea to produce DNA interstrand crosslinks not observed in cells treated with chloroethylnitrosourea alone. *Carcinogenesis.*, **4**, 759.

ZLOTOGORSKI, C., & ERICKSON, L.C. (1984). Pretreatment of human colon tumor cells with the DNA methylating agents inhibits their ability to repair chloroethyl monoadducts. *Carcinogenesis.*, **5**, 83.